# Bidirectional interactions between beet armyworm and its host in response to different fertilization conditions

**DOI:** 10.1371/journal.pone.0190502

**Published:** 2018-01-02

**Authors:** Sifang Wang, Tianbo Ding, Manlin Xu, Bin Zhang

**Affiliations:** 1 Key Lab of Integrated Crop Pest Management of Shandong, College of Plant Health and Medicine, Qingdao Agricultural University, Qingdao, Shandong, the People Republic of China; 2 Shandong Peanut Research Institute, Qingdao, Shandong, the People Republic of China; Chinese Academy of Agricultural Sciences Institute of Plant Protection, CHINA

## Abstract

Fertilizer with different ratios of nitrogen (N) to phosphorus (P) can influence crop plant performance and defense against herbivores. *Spodoptera exigua* is an important agricultural pest that has caused serious economic loss, especially in recent decades. In the present study, we explored effects of different intensities and durations of *S*. *exigua* herbivory on host plant biomass and on *S*. *exigua* enzyme activities in response to five fertilizer treatments with different N: P ratios of 1: 5, 1: 3, 1: 1, 3: 1 and 5: 1. The results showed that fertilizer type can significantly influence interactions between caterpillars and its hosts. Compensatory growth of leaf biomass was detected under fertilizer with N: P = 3: 1. Fertilizer with a higher proportion of N appears to maintain stem biomass in defoliated seedlings similar to controls that are not exposed to herbivory. There was no significant difference in root biomass under most conditions. High proportion of N also enhanced the activity of two antioxidant enzymes, catalase (CAT) and superoxide dismutase (SOD) in low density of beet armyworm. However, with increased herbivorous intensity, a higher proportion of P played a more important role in increasing the activities of CAT and SOD. Higher P likely enhanced acetylcholine esterase (AChE) activity at lower degrees of defoliation, but a higher N proportion resulted in higher AChE activity at higher degrees of defoliation. Higher N proportion contributed to reduced carboxylesterase (CarE) activity at high intensity, short-term defoliation. However, when defoliation intensity increased, the difference in CarE activity between fertilizer categories was little. The study explored the interaction between the damage of *S*. *exigua* and the biomass accumulation of its host plant *Brassica rapa*, and the influence of the N/P ratio in plant fertilizer on this interaction. Systematic analysis was provided on the biomass of *B*. *rapa* and the activity of metabolic enzymes of *S*. *exigua* under different treatments.

## Introduction

Many aspects of herbivorous insect-plant interactions have been studied substantially, such as co-evolution of plants with insects, plant chemistry, insect physiology, behavior and ecology [1]. When insect herbivores are faced with diverse food choices, they often make foraging decisions based on trophic relationships [2]. Many studies have proved that suboptimal diet is of disadvantageous to Lepidopteran by altering their foraging decisions to affect survivorship, growth, development and fecundity [3–5]. Meanwhile, plants have developed several resistant mechanisms to respond to herbivore damage. Compensatory growth is one of the most important resistance mechanism that help plants recover from herbivore damage with the same or more biomass than undamaged plants [6–9].

Nutrients, particularly nitrogen (N) and phosphorus (P) [10], limit plants growth in many ecosystems, and many species can evolve mechanisms to obtain more nutrients or avoid nutrient loss to adapt to different nutritional conditions [11,12]. N and P supply causes up to a 50-fold variation in biomass N: P ratios and is associated with differences in root allocation, nutrient uptake, biomass turnover and reproductive output [13]. N: P ratios in plants (including algae) vary considerably more than in animals or bacteria [13], and much of that variation reflects the relative supply of N and P in the environment [14]. Varying degrees of defoliation by herbivores frequently occur on aquatic and terrestrial plant species [15]. It has been hypothesized that there is a physiological trade-off between growth and secondary metabolism. Thus, fertilizer may actually decrease plant resistance by reducing the amount of secondary metabolites [16]. Many studies have documented that fertilization enhances the growth, development, fecundity, survival, and/or density in several kinds of plant pests [17] such as caterpillars [18,19], leaf beetles [20,21], leaf miners [22,23] and browsing mammals [24,25]. Some other studies have found differently that fertilization enhances or has little effect on pest resistance [26–29]. Thus, the widely accepted paradigm that fertilization enhances insect resistance is not true in all cases.

Beet armyworm, *Spodoptera exigua* (Hübner) (Lepidoptera: Noctuidae), is a worldwide agricultural pest, often causing a serious economic loss. *S*. *exigua* is a polyphagous herbivore that can feed on as many as 130 host plants, e.g. corn, cotton, beet, maize, soybeans, tomato, cabbage, and alfalfa represented over 30 different families [30–32]. As a generalist caterpillar species, *S*. *exigua* have developed some flexible metabolic strategies to nutritional imbalance than mono- and oligophagous insects [33–36]. Most previous studies involved nutrient utilization and performance in a certain Lepidopteran caterpillar were unhesitatingly limited to a defined artificial diet or affected by N variation that provided to its host plants [33,37–40]. However, few studies focused on how the N: P ratios affect *S*. *exigua* performance.

The objectives of this study were (1) to determine the magnitude of compensatory growth for *Brassica rapa* (*campestris*) L. with varying intensities and durations of beet armyworm caterpillars feeding under different N: P fertilizer treatments, and (2) to assess the catalase (CAT), superoxide dismutase (SOD), acetylcholine esterase (AChE) and carboxylesterase (CarE) activities in beet armyworm feeding on seedlings that treated with various levels of fertilization. We hypothesize that feeding process will induce an enhanced/decreased enzyme activities, and the results will clarify a plant response to different herbivorous pressures under varying levels of resources.

## Materials and methods

### Insect rearing and host plant

Beet armyworm eggs were collected from an onion farmland in Jiaozhou, Qingdao, China. When brought in the laboratory and till hatched, larva was reared on an artificial diet in a growth chamber (Shanghai Yiheng Instruments, China) (14 h L: 10 h D photoperiod; 25 ± 1°C; 40–60% RH). In the present study, *B*. *rapa* was selected as beet armyworm’s host plant. Rape seedlings were grown in nursery plates (width: 280 mm, length: 540 mm, depth: 50 mm) in a greenhouse (18 h L: 6 h D photoperiod; 25 ± 1°C), with each containing 50 plugs (each plug is 45 mm in side length of the mouth square and 20 mm in side length of the bottom square).

### Experimental design

Four-week-old seedlings were randomly divided into five groups of 15 plants, and each group was assigned to one of five fertilization treatments (different N: P ratios: N: P = 1: 5, N: P = 1: 3, N: P = 1: 1, N: P = 3: 1 and N: P = 5: 1). According to a certain proportion, ammonium nitrate (AN), monoammonium phosphate (MP) and potassium sulfate (PS) were made up the mother liquid with 25% of mass percent concentration, which included N: P = 1: 5 (contained 0.00 g AN, 41.76 g MP, 20.74 g PS), N: P = 1: 3 (contained 8.30 g AN, 34.00 g MP, 20.74 g PS), N: P = 1: 1 (contained 24.50 g AN, 17.00 g MP, 20.74 g PS), N: P = 3: 1 (contained 34.83 g AN, 6.93 g MP, 20.74 g PS) and N: P = 5: 1 (contained 37.42 g AN, 4.34 g MP, 20.74 g PS) respectively. Additionally, we also prepared microelement mother liquid with 3.332 g micronutrient fertilizer (Ultrasolmicro Rexene APN, Qingdao Sobel Crop Nutrition Co., Ltd., Qingdao, China) and 200 ml H_2_O. When we applied fertilizer, 1500 ml H_2_O, 3 ml mother liquid and 0.75 ml microelement mother liquid were mixed as the fertilizer prepared for plants. All seedlings were maintained under an 18-h photoperiod at 25 ± 1°C in a greenhouse with daily watering and fertilized for 2 weeks. The seedlings from the each group were exposed to an orthogonal combined beet armyworm feeding treatments, namely 5 fertilization × 3 defoliation intensities × 3 defoliation durations. Specifically, 3 defoliation intensities: control (no larva on the seedlings), non-outbreak (two 3^rd^–4^th^ instar larvae on each seeding) and outbreak (five 3^rd^–4^th^ instar larvae on each seeding), and 3 defoliation durations: non-feeding (control), two days and five days feedings were designed in this study. After treatment, beet armyworm larvae were immediately frozen in liquid N and then stored in a −80°C freezer (Thermo Fisher Scientific, Marietta, OH, USA) for subsequent enzyme assays. In addition, one week after beet armyworms were removed, the seedlings in each treatment group were harvested. Harvesting one week after feeding ended allowed us to capture the effects of defoliation on biomass. Immediately following harvest, the leaves, stems and roots (soil medium was rinsed from the roots) were separated and oven-dried at 70°C for 48 h, and weighed to estimate dry biomass.

### Enzyme assays

The activities of two antioxidant enzymes including catalase (CAT) and superoxide dismutase (SOD), and two detoxification enzymes, specifically carboxylesterase (CarE) and acetylcholine esterase (AChE), in 3^rd^ instar caterpillars were assayed to detect changes in metabolism. CarE activity was determined using the method of Van Asperen [41]. CAT, SOD and AChE activities were assayed according to the directions of Reagent Kits (Nanjing Jiancheng Ltd. Co., Nanjing, Jiangsu Province, China). Enzyme activities are expressed relative to protein concentration, which was determined with the Bradford method using bovine serum albumin (Nanjing Jiancheng Ltd. Co., Nanjing, Jiangsu Province, China) as the standard [42].

### Data analysis

Significant differences in leaf, stem and root dry weights and enzyme activities between treatments were identified using three-way ANOVA (Fertilization × Defoliation intensity × Defoliation duration) based on the General Linear Model. In all cases, normality and equal variance tests were performed to verify that ANOVA requirements were satisfied. When significant differences were detected, pairwise multiple comparison procedures (Holm-Sidak method) were performed between and within treatments and treatment levels. All procedures were performed with the statistical software SPSS 19.0 (SPSS Software Inc., Chicago, IL, USA).

## Results

### Leaf biomass

**Fig 1:**
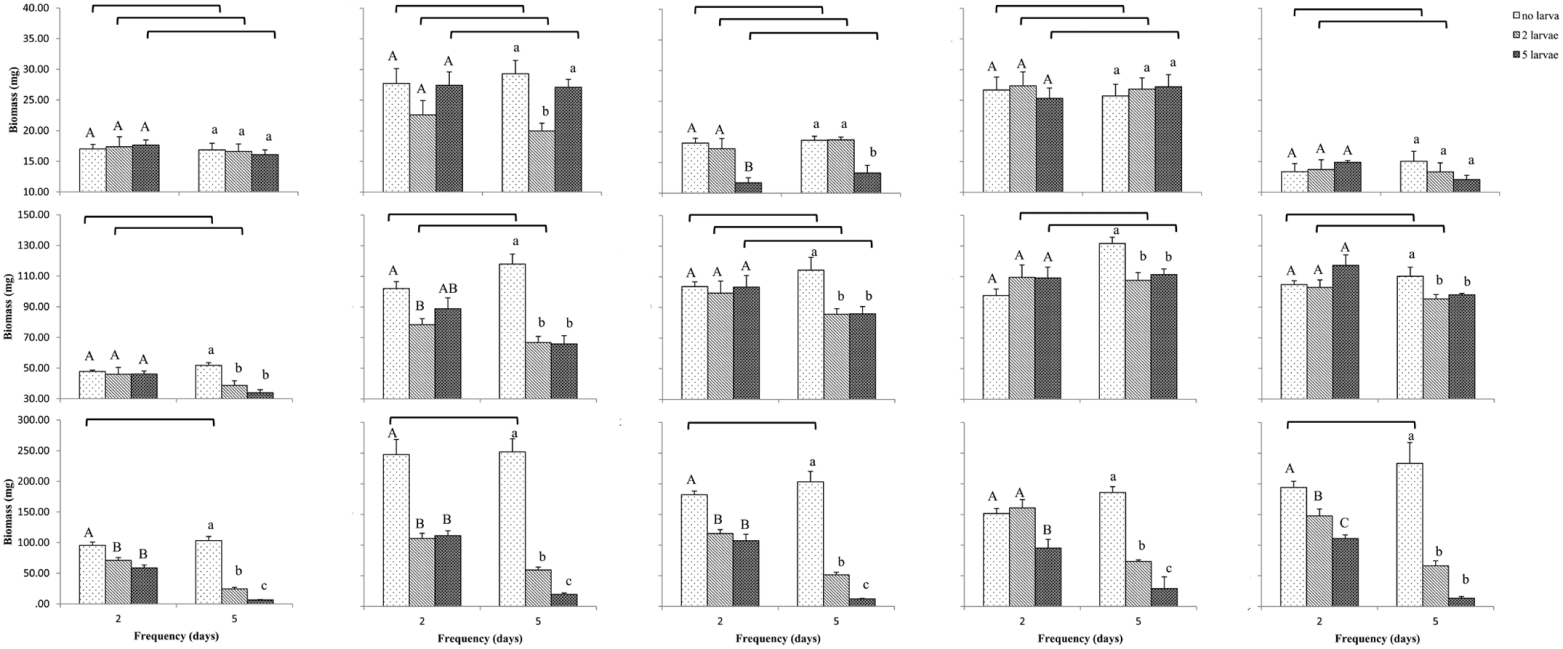
Root, stem and leaf biomass of rape seedlings subjected to herbivory by *Spodoptera exigua* caterpillars. Their biomass were determined 1 week after seedlings were subjected to 2 or 5 days of different intensity herbivory under five fertilization treatments with different ratios of nitrogen to phosphorous: 1: 5, 1: 3, 1: 1, 3: 1 and 5: 1 successively from left column to right column. The five bar charts in first row show root biomass, the five bar charts in second row show stem biomass, the five bar charts in third row show leaf biomass. Bracket line connecting two bars above each bar chart represents no significant difference between the two feeding duration with same intensity herbivory. Same upper letter or same lower letter in each bar chart represents no significant difference between three intensities with same feeding duration.

ANOVA results showed that leaf dry biomass were significantly affected by the fertilization (*F* = 36.77, *df* = 4, *P* < 0.001), defoliation duration (*F* = 95.29, *df* = 1, *P* < 0.001) and intensity (*F* = 316.72, *df* = 2, *P* < 0.001). Significant interactions were found between intensity and fertilization (*F* = 10.57, *df* = 8, *P* < 0.001), and between intensity and defoliation duration (*F* = 54.90, *df* = 2, *P* < 0.001). However, no significant interaction was detected between fertilization and defoliation duration (*F* = 0.59, *df* = 4, *P* = 0.670), and between intensity, fertilization and duration (*F* = 1.47, *df* = 8, *P* = 0.176). Specifically, two days low-density feeding induced a significantly decreased leaf dry biomass in all fertilization levels except for N: P = 3: 1 (*F* = 0.34, *df* = 1, 8, *P* = 0.577); two days high-density feeding caused a dramatically decreased leaf dry biomass under all of fertilization levels. The effects of five days defoliation led to a much more negative effect on leaf dry mass. In addition, there was no significant difference in leaf dry biomass between low-density and high-density under N: P = 1: 5, N: P = 1: 3 and N: P = 1: 1. With increased defoliation duration and intensity, the leaf dry biomass was significantly decreased in comparison to non-feeding rape seedlings (Fig 1).

### Stem biomass

Stem dry biomass was significantly affected by the defoliation intensity and fertilization (*F* = 25.56, *df* = 2, *P* < 0.001; *F* = 170.20, *df* = 4, *P* < 0.001; respectively), however, it was not influenced by defoliation duration (*F* = 2.33, *df* = 1, *P* = 0.130). Significant interactions were detected between intensity and fertilization (*F* = 4.62, *df* = 8, *P* < 0.001), between defoliation intensity and duration (*F* = 21.76, *df* = 2, *P* < 0.001), between fertilization and defoliation duration, and (*F* = 3.78, *df* = 4, *P* = 0.006), however, significant interaction was not found between intensity, fertilization and duration (*F* = 0.72, *df* = 8, *P* = 0.670). Under N: P = 1: 5 level, rape seedlings had the lowest stem biomass, especially at high-density feeding for five days (Fig 1). Like leaf biomass, the stem biomass was also decreased with increased defoliation duration and intensity (Fig 1).

### Root biomass

Root dry biomass was significantly affected by the defoliation intensity and fertilization (*F* = 3.31, *df* = 2, *P* = 0.040; *F* = 87.36, *df* = 4, *P* < 0.001; respectively), however, it was not influenced by defoliation duration (*F* = 0.03, *df* = 1, *P* = 0.868). Significant interactions were only detected between intensity and fertilization (*F* = 4.73, *df* = 8, *P* < 0.001). Without being suffered from feeding, the root dry biomass fertilized by N: P = 1: 3 and N: P = 3: 1 was significantly higher than other three fertilization levels (Fig 1), which suggested that those two ratios enhanced compensation growth after defoliation by beet armyworm caterpillars. Under N: P = 1: 1, significant decreased root biomass was only detected in the higher defoliation duration and intensity levels (*F* = 8.85, *df* = 2, 12, *P* = 0.004; Fig 1).

### Acetylcholine esterase enzyme activity of beet armyworm caterpillar

**Fig 2:**
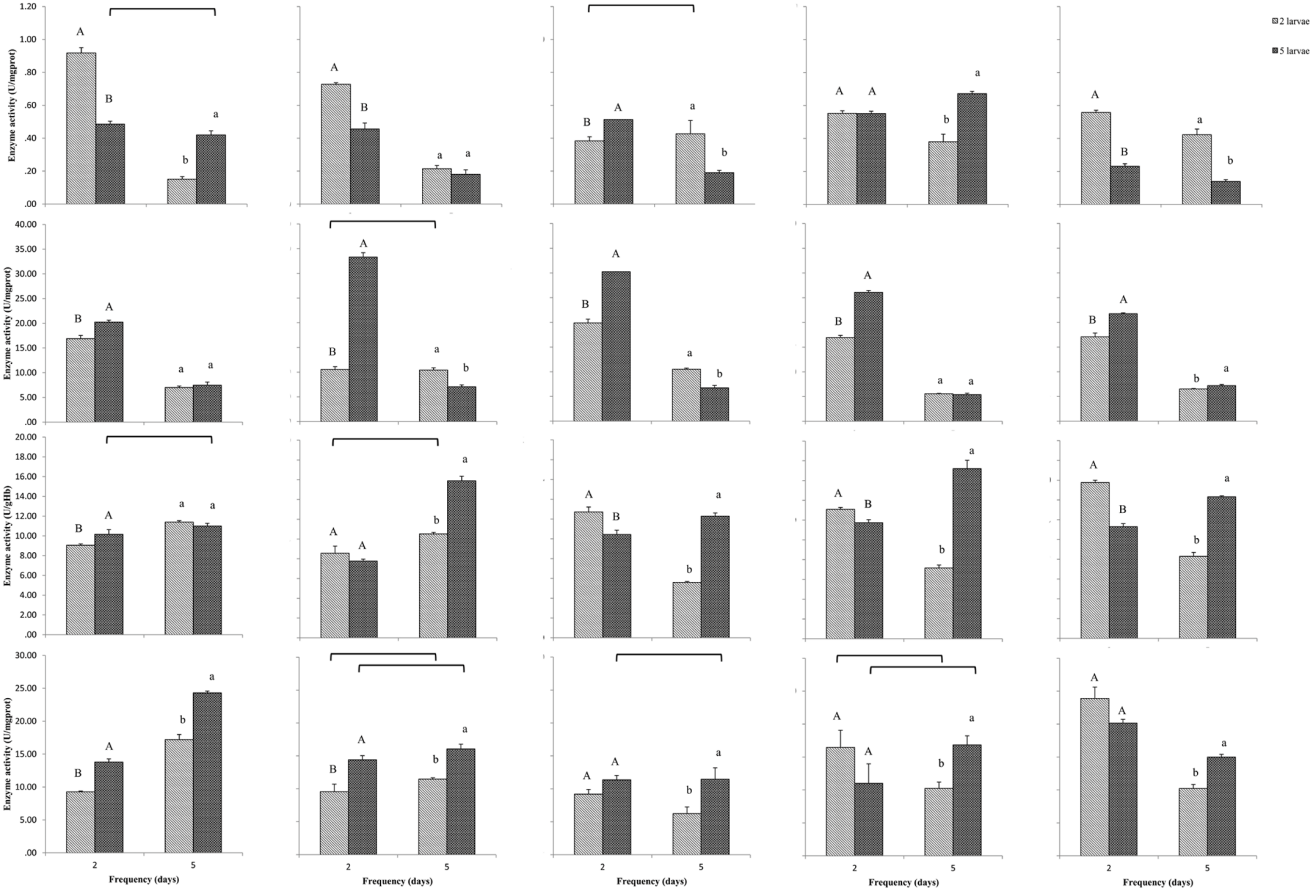
AChE, CarE, CAT and SOD of *Spodoptera exigua* caterpillars which fed on rape seedlings for 2 or 5 days under five fertilization treatments with different ratios of nitrogen to phosphorous: 1: 5, 1: 3, 1: 1, 3: 1 and 5: 1 successively from left column to right column. The five bar charts in first row show AChE activity, the five bar charts in second row show CarE activity, the five bar charts in third row show CAT activity, the five bar charts in forth row show SOD activity. Bracket line connecting two bars above each bar chart represents no significant difference between the two feeding duration with same intensity herbivory. Same upper letter or same lower letter in each bar chart represents no significant difference between three intensities with same feeding duration.

AChE enzyme activity was significantly affected by fertilization, defoliation duration and intensity (*F* = 33.70, *df* = 4, *P* < 0.001; *F* = 283.85, *df* = 1, *P* < 0.001; *F* = 48.03, *df* = 1, *P* < 0.001; respectively). Significant interactions were also detected between intensity and fertilization (*F* = 31.94, *df* = 4, *P* < 0.001), between intensity and duration (*F* = 49.21, *df* = 1, *P* < 0.001), between fertilization and duration (*F* = 37.12, *df* = 4, *P* < 0.001), and between intensity, fertilization and duration (*F* = 45.01, *df* = 4, *P* < 0.001). After two days of low-density defoliation, the AChE enzyme activity was highest in caterpillars that fed on seedlings in the N: P = 1: 5 fertilizer group and was lowest in caterpillars that fed on seedlings in the N: P = 1: 1 fertilizer group (*F =* 95.25, *df* = 4, 20, *P <* 0.001; Fig 2). After five days of high-density defoliation, caterpillars that fed on seedlings fertilized with N: P = 3: 1 had the highest AChE enzyme activity (*F* = 34.64, *df* = 4, 20, *P* < 0.001; Fig 2).

### Carboxylesterase enzyme activity of beet armyworm caterpillars

CarE enzyme activity was significantly affected by fertilization, defoliation duration and intensity (*F* = 51.72, *df* = 4, *P* < 0.001; *F* = 4373.80, *df* = 1, *P* < 0.001; *F* = 439.57, *df* = 1, *P* < 0.001; respectively). Significant interactions were detected between intensity and fertilization (*F* = 43.30, *df* = 4, *P* < 0.001), between intensity and duration of defoliation (*F* = 22.74, *df* = 1, *P* < 0.001), between fertilization and duration of defoliation (*F* = 721.60, *df* = 4, *P* < 0.001), and between intensity, fertilization and duration of defoliation (*F* = 100.62, *df* = 4, *P* < 0.001). Specifically, significantly higher CarE enzyme activity was observed in the two-day defoliation caterpillars other than five-day defoliation ones, especially when they were in high-density (Fig 2).

### Catalase activity of beet armyworm caterpillars

CAT activity was dependent on fertilization and defoliation intensity (*F* = 27.70, *df* = 4, *P* < 0.001 and *F* = 123.50, *df* = 1, *P* < 0.001, respectively), but not affected by defoliation duration (*F* = 2.98, *df* = 1, *P* = 0.088). Significant interactions were also detected between intensity and fertilization (*F* = 15.23, *df* = 4, *P* < 0.001), between intensity and duration of defoliation (*F* = 392.35, *df* = 1, *P* < 0.001), and between fertilization and duration of defoliation (*F* = 61.36, *df* = 4, *P* < 0.001). Furthermore, there was also a significant interaction between intensity, fertilization and duration of defoliation (*F* = 42.09, *df* = 4, *P* < 0.001). CAT activity in caterpillar that fed for five-days defoliation was significantly higher than that was detected in two-days defoliation, especially when the rape seedlings fertilized with lower N: P ratios (Fig 2).

### Superoxide dismutase enzyme activity of beet armyworm caterpillars

Overall, SOD activity in beet armyworm caterpillar was significantly affected by fertilization and intensity (*F* = 23.00, *df* = 4, *P* < 0.001; *F* = 29.43, *df* = 1, *P* < 0.001; respectively) but was not influenced by defoliation duration (*F* = 0.00, *df* = 1, *P* = 0.991). Significant interactions were also detected between intensity and fertilization (*F* = 3.68, *df* = 4, *P* = 0.008), between intensity and duration of defoliation (*F* = 21.12, *df* = 1, *P* < 0.001), between fertilization and duration of defoliation (*F* = 28.04, *df* = 4, *P* < 0.001), and between intensity, fertilization and duration of defoliation (*F* = 3.90, *df* = 4, *P* = 0.006). It should be noted that the significantly enhanced SOD activity was observed when high-density caterpillar fed on rape seedlings that fertilized with N: P = 1: 5 and when low-density caterpillar fed on rape seedlings that fertilized with N: P = 5: 1 (Fig 2).

## Discussion

The present study indicated that fertilizer application, defoliation intensity and duration might mediate the leaf biomass of rape seedlings. After defoliation by beet armyworm caterpillars for two days, there was complete compensation of rape seedlings growth in the N: P = 3: 1 fertilizer group (i.e., leaf biomass was the same in defoliated and non-defoliated seedlings). However, overcompensation (i.e., leaf biomass was higher in the defoliated than in the non-defoliated seedlings) was not observed in any other treatment groups. These results demonstrated that N: P = 3: 1 fertilization level and low defoliation intensities could modulate a compensatory growth in rape plants, and these effect could be increased with an increasing ratio of N to P. However, an excessive proportion of N did not result in overcompensation, which was demonstrated by the fact that the leaf dry biomass was the same for all seedlings fertilized with N/P = 5/1.

Previous studies widely approved that the compensatory growth occurred only in resource-rich environments [43–48]. A few studies have reported that defoliation duration plays a much more important role than resources in modulating compensatory growth at a relatively low intensity of defoliation; however, at high defoliation intensities resource availability does more [48,49]. In the current study, low-intensity defoliation treatment (feeding by two caterpillars for two days) might have been too high of the magnitude for overcompensation growth to occur. We conclude that compensatory growth of rape leaf was associated with the ratio of N to P in fertilizer.

Although the rape stem is located above the ground, it is usually not damaged by beet armyworm caterpillars. With two days of low- or high-intensity defoliation, there was no significant difference in stem biomass between defoliated and control seedlings, except for a lower stem biomass in low-intensity defoliated seedlings. With five days of low- or high-intensity defoliation, stem biomass was lower in defoliated seedlings than in control seedlings, except for the N: P = 5: 1 fertilizer group, in which there was no significant difference in stem biomass between defoliated and control seedlings. This result indicated that stem biomass was not influenced by low-level defoliation but significantly decreased after high-level defoliation under almost all fertilizer treatments. It is worth noted that a higher N proportion appears to contribute to maintaining stem biomass in defoliated seedlings. Usually, stems are not the feeding target of *S*. *exigua*, but when the intensity of herbivory increased, part of the stem was also eaten by caterpillars. In this case, higher N in fertilizer seems to compensate for biomass lost by defoliation.

Located underground, roots cannot be directly damaged by caterpillars. With two or five days of low-intensity defoliation, there was no significant difference in root biomass between defoliated and control seedlings, except for seedlings exposed to low-intensity defoliation for 5 days in the N: P = 1: 3 fertilizer group. When the host plants were exposed to high-intensity defoliation, there was only a significant difference in root biomass between defoliated and control seedlings within N: P = 1: 1 fertilizer group. This result indicates that root biomass might not be influenced by the low-level defoliation, but significantly decreased after high-level defoliation in fertilizers containing specific ratios of N to P.

In woody plants, after defoliation, translocation of photoassimilates from foliage to roots is severely reduced, which in turn reduces root growth. Meanwhile, as new stem buds are activated to produce new leaves, reserves are translocated from the roots to promote active growth in these carbohydrate sinks, which further compromises root growth [48]. In the present study, most fertilizer applications could meet the requirements of the herbage plant rape after low-level defoliation. However, growth was not compensated under a limited fertilizer, which resulted in a decrease in root biomass after high-level defoliation.

AChE is an enzyme that breaks down the neurotransmitter acetylcholine at the synaptic cleft as a result the next nerve impulse can be transmitted across the synaptic gap [50]. At low-intensity, AChE activity increased with increasing phosphorus in the fertilizer. After five days of defoliation, the lowest AChE activity was detected in fertilizer with the lowest proportion of N. At high-intensity, the highest AChE activity was observed within caterpillars that fed on plants fertilized by N: P = 3: 1. The results demonstrated that at lower degrees of defoliation (low density and short term), higher P likely contributes to enhanced AChE activity, and higher N proportions may result in higher AChE activity at high-intensity of defoliation (high density and long term).

CarE is a metabolic enzyme involved in insecticide resistance [51], allelochemical metabolism and tolerance [51], cell-to-cell interaction [52], pheromone degradation [53] and hydrolysis of the neurotransmitter acetylcholine and juvenile hormone (JH) [54,55]. After two days of low-intensity defoliation, the highest CarE activity was detected in caterpillars that fed on plants fertilized by N: P = 1: 1. These results demonstrated that the host plants treated with N: P = 1: 1 fertilizer might be prone to generating more effective plant allelochemicals that are toxic to *S*. *exigua* when exposed to low-intensity defoliation for two days. The hypothesis could be based on the conclusions of some previous study [56,57] With the extended defoliation duration (five days), the highest activity was found in N: P = 1: 1 and N: P = 1: 3 treatment groups. Interestingly, the lowest CarE activity was observed in caterpillars that fed on seedlings fertilized with N: P = 1: 3 that experienced 2 days of low-intensity defoliation. This result indicated that the effects of N: P = 1: 3 fertilizer on CarE activity are mediated by the duration of defoliation. After two days of high-intensity defoliation, the lowest CarE activity was observed in caterpillars that fed on seedlings supplied with fertilizer that had the highest proportion of N and the highest proportion of P. CarE activity increased with the increase in P proportion. After five days of high-intensity defoliation, CarE activities of caterpillars that fed on seedlings in different fertilizer groups were almost similar, except the CarE activity in the N: P = 3: 1 group was significantly lower. These results indicate that higher N content contributed a lot to the decreased CarE activity in high-intensity and short-term defoliation. However, when the defoliation intensity increased, the difference in CarE activity among fertilizer groups was small. We concluded that the feeding process can influence the activities of a suite of enzymes that are related to defenses against adverse environments.

SOD and CAT are important antioxidant enzymes that protect cells from the damaging effects of oxidative radicals [58]. In this study, significant differences in SOD and CAT activity were found between different groups, indicating fertilization condition could mediate the antioxidant defenses in caterpillars. With the increased intensity of defoliation, significantly enhanced antioxidant enzymes were detected in caterpillars that fed on rape seedlings supplied with fertilizer with the lowest proportions of N. The lowest SOD and CAT activities were observed for fertilizer with equal N to P. These results indicate that fertilizer with a high proportion of N contributes to enhance the host plant defense against herbivorous insects. However, with the extension of feeding time, a high proportion of P applied to the host plants played a more critical role in inducing higher antioxidant enzyme activity in beet armyworms. When herbivorous insects ingest pro-oxidant allelochemicals, they intend to eliminate increased oxidative stress by enhancing antioxidant enzyme activity [59]. Therefore, the high proportion of N in a fertilizer could contribute a lot to the generation of pro-oxidant allelochemicals by the host plant to defend against herbivore attack under low-intensity defoliation. However, when the herbivorous intensity increased, a higher proportion of P in the fertilizer played a more important role in host plant defense against foraging beet armyworms.

In conclusion, our result indicated that N to P ratios can significantly influence the interaction between host plants and caterpillars. This interaction can be owing to a compensatory growth, depending on fertilizer levels. Fertilizer with a high proportion N appears to enhance stem biomass in defoliated seedlings. Seedlings treated with high proportion in N also contributed to enhance the activity of CAT and SOD in its beet armyworm at low density. High P proportion enhanced AChE activity at lower defoliation, but high N proportion results in high AChE activity at higher defoliation. The results in the present study will contribute to revealed the relationship between herbivorous insects and plants under different nutritional conditions and provide the basic data for correcting the cropping system and economic threshold.

## Supporting information

S1 Table Annotated experimental design. Annotated experimental design for evaluating how compensatory plant growth operates under different intensities and durations of rape seedling defoliation as well as under five fertilizer treatments. The number of replicates is 5 for all treatments.
(DOC)

S2 Table Summary of the results of statistical analyses for experiments testing the effects of fertilization as well as the duration and intensity of defoliation by beet armyworm caterpillars on rape seedling leaf, stem and root biomass. (DOC)

S3 Table Summary of the results of statistical analysis for experiments testing the effects of defoliation duration and intensity on leaf and root biomass. Notes.
(DOC)

S4 Table Summary of the results of statistical analyses for experiments testing the effects of fertilization as well as the duration and intensity of defoliation by beet armyworm caterpillars on the activities of two antioxidant enzymes (superoxide dismutase, SOD and catalase, CAT) and two detoxification enzymes (carboxylesterase, CarE and acetylcholinesterase, AChE). (DOC)
