# Supplementary material for: Bidirectional interactions between beet armyworm and its host in response to different fertilization conditions
Source: PLoS One. 2018 Jan 2;13(1):e0190502. doi: 10.1371/journal.pone.0190502 (PMC5749815; doi:10.1371/journal.pone.0190502)
Supplement: S1 Table — Annotated experimental design for evaluating how compensatory plant growth operates under different intensities and durations of rape seedling defoliation as well as under five fertilizer treatments. The number of replicates is 5 for all treatments. (DOC) [file pone.0190502.s001.doc]

S1 Table

| Fertilization (ratio of nitrogen to phosphorus) | 1 : 5 | | | | 1 : 3 | | | | 1 : 1 | | | | 3 : 1 | | | | 5 : 1 | | | |
| --- | --- | --- | --- | --- | --- | --- | --- | --- | --- | --- | --- | --- | --- | --- | --- | --- | --- | --- | --- | --- |
| Intensity Level (number of caterpillars per seedling) | 2 | | 5 | | 2 | | 5 | | 2 | | 5 | | 2 | | 5 | | 2 | | 5 | |
| Defoliation Duration (number of days of feeding) | 2 | 5 | 2 | 5 | 2 | 5 | 2 | 5 | 2 | 5 | 2 | 5 | 2 | 5 | 2 | 5 | 2 | 5 | 2 | 5 |
