# Supplementary material for: Bidirectional interactions between beet armyworm and its host in response to different fertilization conditions
Source: PLoS One. 2018 Jan 2;13(1):e0190502. doi: 10.1371/journal.pone.0190502 (PMC5749815; doi:10.1371/journal.pone.0190502)
Supplement: S2 Table — (DOC) [file pone.0190502.s002.doc]

S2 Table

| Source | Low intensity (2 caterpillars per plant) | | |  | High intensity (5 caterpillars per plant) | | |
| --- | --- | --- | --- | --- | --- | --- | --- |
|  | Root  (F-ratio and P-value) | Stem  (F-ratio and P-value) | Leaf  (F-ratio and P-value) |  | Root  (F-ratio and P-value) | Stem  (F-ratio and P-value) | Leaf  (F-ratio and P-value) |
| Defoliation Duration (DD) | ～ | 6.91^*^ | 198.33^**^ |  | ～ | 17.68^**^ | 216.46^**^ |
| Fertilization (F) | 19.02^**^ | 53.45^**^ | 25.34^**^ |  | 57.80^**^ | 60.42^**^ | 4.96^**^ |
| DD×F | ～ | ～ | 2.85^*^ |  | ～ | ～ | 2.87^*^ |

Notes.

The significance of fixed factors was determined with F-test statistics. Only significant interactions are reported, and main effects or their interactions are marked ～ when they are not significant.

Significance is reported as:

^**^ *P* < 0.001.

^*^ *P* < 0.05.
