# Supplementary material for: Bidirectional interactions between beet armyworm and its host in response to different fertilization conditions
Source: PLoS One. 2018 Jan 2;13(1):e0190502. doi: 10.1371/journal.pone.0190502 (PMC5749815; doi:10.1371/journal.pone.0190502)
Supplement: S3 Table — Notes. (DOC) [file pone.0190502.s003.doc]

S3 Table

| Defoliation Duration | Low intensity (2 caterpillars per plant) | | |  | High intensity (5 caterpillars per plant) | | |
| --- | --- | --- | --- | --- | --- | --- | --- |
|  | Root  (F-ratio and P-value) | Stem  (F-ratio and P-value) | Leaf  (F-ratio and P-value) |  | Root  (F-ratio and P-value) | Stem  (F-ratio and P-value) | Leaf  (F-ratio and P-value) |
| Two days | 7.64^*^ | 17.92^**^ | 13.87^**^ |  | 25.27^**^ | 19.22^**^ | 6.28^*^ |
| Five days | 22.84^**^ | 67.49^**^ | 4.86^*^ |  | 34.20^**^ | 66.68^**^ | ～ |

Notes.

Fixed factors are tested with F-test statistics. Only significant interactions are reported with main effects or their interactions marked ～ removed because they were not significant.

Significance is reported as:

^**^ P < 0.001.

^*^ P < 0.05.
