# Supplementary material for: Bidirectional interactions between beet armyworm and its host in response to different fertilization conditions
Source: PLoS One. 2018 Jan 2;13(1):e0190502. doi: 10.1371/journal.pone.0190502 (PMC5749815; doi:10.1371/journal.pone.0190502)
Supplement: S4 Table — (DOC) [file pone.0190502.s004.doc]

S4 Table

| Source | Low intensity (2 caterpillars per plant) | | | |  | High intensity (5 caterpillars per plant) | | | |
| --- | --- | --- | --- | --- | --- | --- | --- | --- | --- |
|  | AChE  (F-ratio and P-value) | CarE  (F-ratio and P-value) | SOD  (F-ratio and P-value) | CAT  (F-ratio and P-value) |  | AChE  (F-ratio and P-value) | CarE  (F-ratio and P-value) | SOD  (F-ratio and P-value) | CAT  (F-ratio and P-value) |
| Defoliation Duration (DD) | 189.22^**^ | 675.24^**^ | 11.56^*^ | 221.83^**^ |  | 97.60^**^ | 5040.22^**^ | 9.71^*^ | 183.56^**^ |
| Fertilization (F) | 3.45^*^ | 25.90^**^ | 16.68^**^ | 20.47^**^ |  | 122.02^**^ | 76.27^**^ | 10.50^**^ | 22.05^**^ |
| DD×F | 42.04^**^ | 42.19^**^ | 22.80^**^ | 103.77^**^ |  | 38.10^**^ | 83.13^**^ | 10.17^**^ | 21.36^**^ |

Notes.

Fixed factors are tested with F-test statistics. Only significant interactions are reported with the main effects or their interactions marked ～ removed because they were not significant.

Significance is reported as:

^**^ P < 0.001.

^*^ P < 0.05.
